# Impact of integrated yoga therapy on cognitive impairment and cardiac dysfunction in relation to quality of life in breast cancer patients undergoing chemotherapy: Study protocol for a two-arm randomized controlled trial

**DOI:** 10.3389/fonc.2022.955184

**Published:** 2022-09-16

**Authors:** Ganagarajan Inbaraj, Talakad N. Sathyaprabha, Kaviraja Udupa, Amritanshu Ram, Shekar Patil, Jamuna Rajeswaran, Krishna K. Nandakumar, Spoorthi Belur, Arman Deep Singh, Parthipulli Vasuki Prathyusha, Sapna K. Bayari, Rao M. Raghavendra

**Affiliations:** ^1^ Department of Neurophysiology, National Institute of Mental Health and Neurosciences, Bangalore, Karnataka, India; ^2^ Department of Complementary and Alternative Medicine, HealthCare Global, Bangalore, Karnataka, India; ^3^ Department of Clinical Oncology, HealthCare Global, Bangalore, Karnataka, India; ^4^ Department of Clinical Psychology, National Institute of Mental Health and Neurosciences, Bangalore, Karnataka, India; ^5^ Centre for Ayurveda Biology and Holistic Nutrition, The University of Trans-Disciplinary Health Sciences and Technologies (TDU), Bengaluru, Karnataka, India; ^6^ Department of Biostatistics, National Institute of Mental Health and Neurosciences, Bangalore, Karnataka, India; ^7^ Central Council for Research in Yoga and Naturopathy, New Delhi, India

**Keywords:** breast cancer, yoga, chemotherapy, cognition, autonomic dysfunction, fMRI, quality of life

## Abstract

**Background:**

Chemotherapy-related cognitive impairment (CRCI) and cardiac dysfunction (CRCD) are common adverse effects seen in breast cancer patients undergoing chemotherapy. Even though these effects significantly influence daily functioning and overall quality of life, effective strategies to avoid and/or mitigate these adverse effects remain elusive. Yoga as a Mind-body intervention has been used increasingly by cancer patients and has undergone empirical investigations as a potential intervention for patients with cancer. Furthermore, yoga is associated with improved cognition and cardiac functioning in healthy older adults and subjects with cognitive and cardiac impairments. Accordingly, in the current study, yoga holds promise as an intervention to prevent/manage CRCI and CRCD with improved overall QOL in women receiving chemotherapy for breast cancer.

**Methods:**

The study is a two-arm, randomized controlled trial. Women diagnosed with stage I-III breast cancer and awaiting neo-adjuvant or adjuvant chemotherapy will be recruited from a tertiary care center in Bangalore, India. Following recruitment, subjects are randomized to the intervention group (integrated yoga therapy intervention during chemotherapy) or the control group (standard care during chemotherapy). The study’s primary outcome is to measure the quality of life (cognitive domain) using European Organization for the Research and Treatment of Cancer Quality of Life Questionnaire (EORTC QLQ-C30). The other primary objectives will include cognitive functioning using neuropsychological test battery and cardiac autonomic function testing using heart rate variability. Secondary outcomes are Brain-derived neurotrophic factor (BDNF), brain function (functional MRI), Echocardiography, serum cortisol, Functional assessment of cancer therapy-cognition (FACT-Cog V3), perceived stress scale and Ryff Scales of Psychological Well-Being. Assessments take place before, during and after chemotherapy; 16-weeks post chemotherapy and 1-year post-baseline.

**Discussion:**

Yoga is a promising intervention for preventing and/or managing chemotherapy-related adverse effects (CRAE) and enhancing the quality of life among breast cancer patients. The findings from this study may also help understand the inner mechanisms involved in the protective and restorative effects of yoga on CRAE and support the use of yoga prophylactically for breast cancer patients. In addition, the results of this study could help chemotherapy-exposed individuals with other solid cancer types who have cognitive and cardiac issues.

**Ethics and Dissemination:**

The study is approved by the ethics committee of the HealthCare Global Enterprises Ltd. Hospital (EC/434/19/01) and National Institute of Mental Health and Neurosciences (NIMH/DO/ETHICS SUB-COMMITTEE (BS&NS) 9th MEETING/2018).

**Clinical Trial Registration:**

http://ctri.nic.in/Clinicaltrials/advancesearchmain.php, identifier CTRI/2020/10/028446.

## Background

Breast cancer is one of the most frequently diagnosed cancers globally, comprising 16% of all cancer types (1, 2). Evidence suggests that chemotherapy used to treat breast cancer causes inadvertent chemotherapy-associated toxicities (3–5). However, more subtle adverse effects, such as potential changes in memory or cognition, have received little attention, despite many women reporting subjective changes in memory and the ability to think clearly during and after chemotherapy. These cognitive deficits, even mild, can cause significant psychological distress and profoundly impact daily functioning and life quality (6, 7). Chemotherapy-related cognitive impairment (CRCI) typically manifests in 75% of patients during chemotherapy (acute effect) and 35% post-chemotherapy (long-term effect)- persist for months or even years after completing chemotherapy (8–10). Multiple cross-sectional and longitudinal studies have shown subjective and objective associations between chemotherapy and cognitive impairments in breast cancer patients and survivors (11–14). In addition, meta-analyses show that women who undergo chemotherapy for breast cancer perform poorly on neuropsychological tests examining attention, executive functioning, working memory, spatial ability, verbal/language ability, and information-processing speed compared to women who have not undergone chemotherapy or controls without a history of cancer (11, 15, 16). The underlying aetiology for these cognitive deficits is unknown but presumed multifactorial and caused by direct and indirect mechanisms leading to adverse structural and functional changes, such as atrophy of cerebral grey matter and white matter demyelination (17–19). In recent years, there has been emerging data on the influence of autonomic nervous system (ANS) on cognitive functioning (20). Numerous studies ascertain that cardiovascular autonomic dysfunction (CAD) attributed to the use of chemotherapeutics called Chemotherapy-related autonomic dysfunction (CRCD) leads to prolonged activation of the hypothalamic-pituitary-adrenal (HPA) axis and the elevated glucocorticoid levels (21–24). These elevated glucocorticoids cause disturbed cardiac function such as arrhythmias, hypertension, systolic dysfunction, myocardial ischemia, thromboembolism, heart failure, cardiomyopathy or other adverse events affecting patient’s quality of life and increasing the chance of cardiovascular mortality (25, 26). The primary underlying pathophysiological mechanism involved in the entire process is believed to be caused by a generalized deficit in the cholinergic function that affects both sympathetic and parasympathetic systems of the ANS (27). On that concern, global markers that help estimate autonomic dysfunction with a prospect of excellent physiological correlate are heart rate variability (HRV) and Left Ventricular Ejection Fraction (LVEF), which could be considered a promising early biomarker of cognitive and cardiac impairment in patients receiving chemotherapy for breast cancer. Since cardiotoxicity can develop during cancer treatment - or - can occur within days, months, or years after cancer treatment, assessing LVEF plays a vital role in assessing the severity of a decrease in the systolic function of the heart and thus helps direct the management of various cardiovascular diseases (28).

All these factors of CRCI, CRCD and psychological distress contribute to increased hospital visits and costs, ultimately leading to lowered quality of life. Since, few available interventions address these chemotherapy-associated adverse (29), there is a need for evidence-based therapeutic interventions to prevent cognitive decline and cardiac dysfunction experienced by breast cancer patients during chemotherapy. Practices such as yoga have shown promising results in improving cognitive (30) and cardiac functioning as studied in subjects with cancer (31). Hence this study aims to evaluate the effects of yoga intervention on CRCI, CRCD and QOL in breast cancer patients undergoing chemotherapy.

Yoga, an ancient Indian science, is widely used all over the globe as a complementary and alternative therapy which has been increasingly used in cancer patients to reduce stress, mood states, distress and improve QOL (32). Results from earlier studies on breast cancer patients have shown yoga to reduce self-reported affective states (anxiety and depression) (33, 34), chemotherapy-induced nausea and emesis (34), improvement in post-operative outcomes such as wound healing (35), reduction in pro inflammatory cytokine TNF alpha (33), improvement in natural killer cell counts (33) and reduction in symptom distress (33). Yoga has also been shown to improve various aspects of quality of life such as cognitive function, physical function and emotional function in breast cancer patients on treatment (30, 36). Besides improving quality of life, yoga also helps reduce genotoxic stress such as DNA damage (34) morning salivary cortisol levels (31) and improve the BDNF levels (37). In breast cancer survivors with advanced metastatic disease, yoga help reduce fatigue, improve sleep and reduce symptom burden (30). Yoga has been shown to enhance QOL, cortisol rhythm, and HRV in women with breast cancer undergoing radiotherapy compared to stretching alone, suggesting that yoga benefits are due to more than simple stretching, social support, or other indirect effects (38). Furthermore, yoga normalizes gastric motility and reduced cardiac autonomic stress arousal in breast cancer patients (39).

Results from these studies suggest yoga has beneficial findings concerning cognitive and cardiac function in cancer patients. Findings from all these studies suggest that inclusion of yoga during chemotherapy should not only bring alleviation from cognitive ill-effects of chemotherapy but also address issues resulting to it like distress, positive and negative psychological effect, and thereby improve quality of life. Thus, we aimed to determine the effect of integrated yoga therapy program on cognition and cardiac function assessed using EORTC QLQ-C30, Neuropsychological test battery, fMRI, blood sampling, HRV, 2D echocardiography and subjective questioners in breast cancer patients receiving chemotherapy. The results expected from the current study could be used as a preventive perspective in minimizing the risk of developing cognitive impairment by subclinical detection of cardiac autonomic dysfunction during chemotherapy and improving overall well-being.

## Methods/design

### Aims and objectives

The overall aim of the study is to examine whether Integrated Yoga Therapy Program (IYTP) can improve cognitive functioning, cardiac functioning, and quality of life in breast cancer patients receiving chemotherapy.

The objectives are as follows:

1. To investigate the effect of IYTP on cognitive impairment in breast cancer patients undergoing chemotherapy.
2. To assess the role of IYTP on functional cognitive domains that affect neural circuitry changes using fMRI and effect of IYTP on treatment-related distress, perceived stress, and quality of life.
3. To assess cardiac autonomic dysfunction using heart rate variability and associate its changes with cognitive performance.
4. To investigate the impact of IYTP on brain-derived neurotrophic factor (BDNF) and cortisol, which in turn has its role on cognition and cardiac function.

### Study design

**Figure 1:**
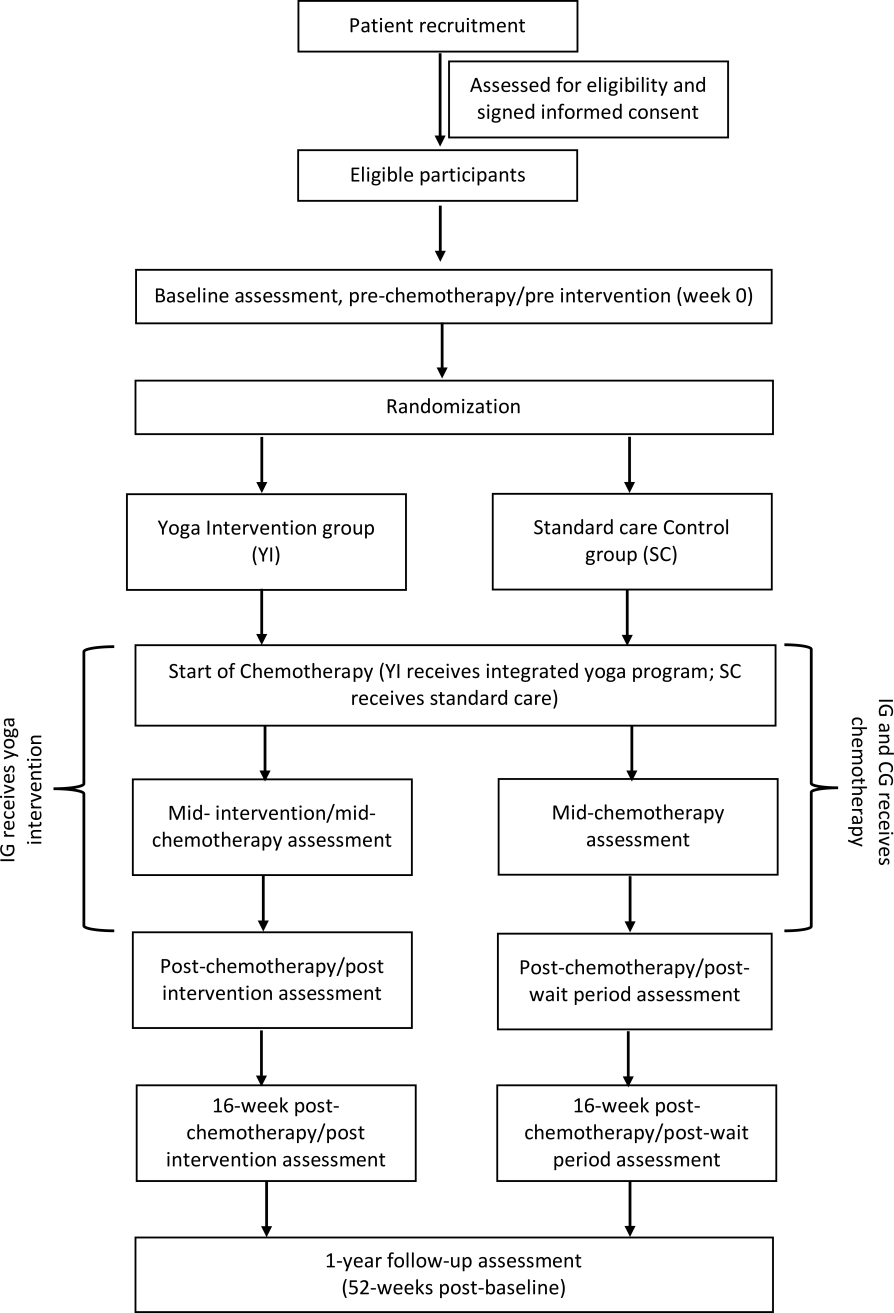
Flow diagram illustrating study methods.

**Figure 2:**
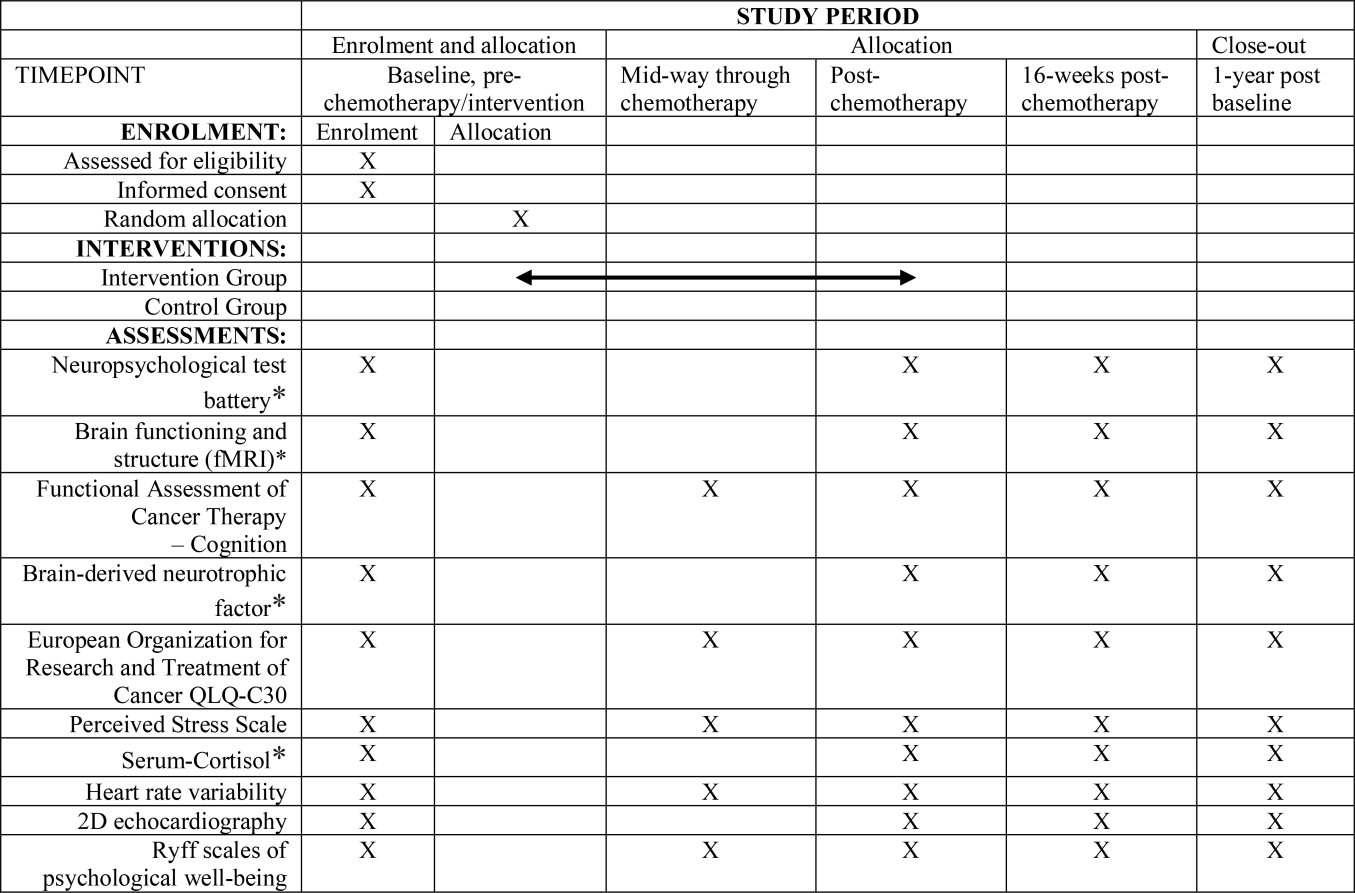
A detailed description of the assessments measures, *assessment is optional at follow-ups. Standard Protocol Items: Recommendations for Interventional Trials (SPIRIT) schedule.

The study is a two-arm, parallel-group Randomised Control Trial. The design adheres to the Standard Protocol Items Recommendations for Interventional Trials (SPIRIT) (40) and the Consolidated Standards of Reporting Trials (CONSORT) (41). After completing a baseline (pre-chemotherapy) assessment, recruited women are randomly assigned to one of two groups: (I) yoga intervention, in which they receive an integrated yoga therapy program during chemotherapy (YI), or; (II) standard care control condition, in which they receive routine care during chemotherapy (SC). Additional evaluations occur mid-way through chemotherapy, post-chemotherapy, 16-weeks post-chemotherapy (first follow-up), and one-year post-baseline (second follow-up). **Figure 1**: outlines the trial design, and **Figure 2**: summarizes the enrolment schedule, intervention and assessment points.

### Recruitment and procedures

Patients diagnosed with stage I-III breast cancer will be recruited from Health Care Global (HCG) Cancer Hospital in Bengaluru, India. Initially, healthcare providers identify the potentially eligible participants and orient the study in brief to them. Patients are then contacted by study staff to confer their eligibility and receive informed consent to participate in the study. Patients are also recruited *via* posters displayed in waiting lobbies and examination chambers, advertisements on the hospital’s webpage, and word-of-mouth promotion. Women asking for more information about the study are encouraged to contact research staff over the phone or by email.

### Participant eligibility

#### Inclusion criteria

Women who meet the following criteria will be eligible to participate in the study: (1) 18–60 years of age; (2) diagnosed with stage I-III (i.e., non-metastatic) breast cancer; (3) scheduled to receive at least six cycles of adjuvant or neoadjuvant chemotherapy; (4) can speak and comprehend English (in order to perform neuropsychological test battery) and; (5) have support from their medical oncologist to participate in the yoga intervention. They must also score ≥23 on the Montreal Cognitive Assessment (MoCA) (42) and ≥ 55 on the LVEF of echocardiography before randomization.

#### Exclusion criteria

Patients who match the following exclusion criteria will not be eligible to participate in the study: (1) prior exposure to chemotherapy or radiotherapy; (2) during screening, a score of ≤23 on the Montreal Cognitive Assessment (MOCA) scale (as a score less than 23 is considered cognitively impaired) (3) diagnosis of neurological disorders such as epilepsy, Parkinson’s disease, neurodegenerative disorders; (4) severe anxiety or mood disorder (e.g., major depressive disorder) diagnosed within the past year; (5) traumatic brain injury or concussion with persisting symptoms (e.g., dizziness, headaches, lack of attention) at the time of screening; (6) above 60 years of age (as cognitive abilities often decline with an increase in age) (7) substance use disorder (e.g., alcohol, narcotics); (8) regular participation in an active behavioral intervention/yoga in last six months. (9) Injuries or illnesses (e.g., orthopedic injury, acute arthritis) that prevent stretching while practising asanas.

#### Added inclusion/exclusion criteria for MRI/fMRI

During the initial screening phase, eligible participants will be asked if they would also be interested in undergoing an additional optional MRI/fMRI evaluation. The MRI/fMRI is performed at three different time points: baseline (before chemotherapy), post-chemotherapy, and 1-year post-baseline. The additional exclusion criteria listed below apply to the MRI/fMIR evaluation: (1) Left-handedness (owing to lateralization of language in right-handed people); (2) Metal implants (such as pacemakers) or metal dental work other than fillings (which are not compatible with MRI/fMRI); (3) claustrophobic; (4) current breast tissue expanders (since these are not MRI/fMRI compatible), and; (6) Lower back ache that would prevent lying motionless for an hour.

#### Screening procedure and informed consent

Initial screening will be performed to ensure that participants meet the primary eligibility requirements. If women are determined to be eligible, MOCA and 2D echocardiography (for evaluating Left Ventricular Ejection Fraction-LVEF) will be administered. MOCA is a quick 30-point test that will be used to assess cognitive deficits, and LVEF is a marker for cardiac health. MoCA and LVEF are administered prior to any study-related activity to establish women’s final eligibility. Patients scoring ≥ 24 on MoCA and ≥ 55 on the LVEF are invited to read and sign the informed consent form.

#### Randomization

Subjects satisfying the selection criteria and consenting to participate will be assessed at baseline with subjective and objective measures prior to starting chemotherapy. Randomization will then be done with an allocation ratio of 1:1 into the yoga (YI) or control (SC) group following the baseline assessment using computer generated random numbers. Allocation concealment will be done by using opaque envelopes with group assignments by personnel not involved in the study. The order of enrolment will be verified using hospital records.

#### Blinding

Participants and study personnel will be naïve to group allocation since randomization occurs after baseline evaluations. Following randomization, a single-blind procedure is used so that the study staff who do the assessments and the statistician who looks at the data will be blinded to group allocation. All patient identifiers will be removed, and data will be analysed blindly. Unblinding will be done only after all data are analysed and entered into the database after completing the study. This would ensure unbiased analyses of the data.

#### Sample size estimation

Considering the effect size observed, in an earlier study by Vadiraja & Rao et al. (31), with improvement in cognitive function domain of EORTC Qol-C30 between yoga and control group as 0.49 with a 95% confidence interval and 80% power, we need a sample size of 67 subjects in each arm. If attrition is within 20% through the course of the intervention, 75 subjects will be recruited in each arm. Therefore 150 consenting subjects will be randomized into each group (yoga, n=75, and controls, n=75) with an allocation ratio of 1:1. The analyses would consider a two-tailed probability of p < 0.05 as significant. This ensures that a statistical type I error (probability of detecting a false positive) is less than 5%. The analyses will involve ANCOVA using the respective baseline measure as a covariate.

### Integrated Yoga Therapy program (IYTP)

The IYTP intervention will be given to participants along with their chemotherapy. For those in the YI group, it starts up to a week before chemotherapy begins and lasts until chemotherapy ends. The intervention is based on earlier protocols employed by the study team for breast cancer patients and found to have good patient adherence (32, 39) that they could perform these practices 5 days/week during intensive chemotherapy treatment. **Table 1** summarizes the IYTP prescription. The intervention will consist of 14 - 24 weeks (five days a week) of hospital and home-based moderate yoga therapy sessions compared with standard care alone while patients undergo chemotherapy. The practices will be taught in the hospital by a trained yoga therapist during chemotherapy and supervised online for home practices. The initial one-week yoga session will be administered in person as a one-to-one session in the hospital setup between 9 AM to 10 AM. After the patient is discharged from the hospital post their first chemotherapy cycle, they will be advised to continue yoga practice at home between 7 AM to 9 AM with a documented video and an intervention brochure outlining the instructions and benefits of yoga. Subsequently, when they come to the hospital for their following chemotherapy cycles (once every 21 days throughout their six cycles of chemotherapy), they will be refreshed with in-person yoga sessions, where necessary refinements to their practices will be made. Also, Also, their performance in yoga will be graded using the “Yoga Performance Assessment” (YPA) scale, a tool devised by Swami Vivekananda Yoga Anusandhana Samsthana (SVYASA) and Advanced Centre for Yoga (NIMHANS) for recording patient compliance to yoga practices (43).

**Table 1:** Showing the list of practices.

	Practice List	Time in minutes
**1.**	**Loosening Practices SukshmaVyayama (4 minutes)**	
1.a	Neck Forward & Backward bending	1
1.b	Neck Side Bending	1
1.c	Neck Rotation – Clockwise and Anti-Clockwise	2
**2**	**Breathing Practices (3 minutes)**	
2.a	Hands In & Out Breathing	1
2.b	Shoulder Stretch Breathing	1
2.c	Ankle Stretch Breathing	1
**3.**	**Yoga Postures or Asanas (7 minutes)**	
3.a	Tadasana (Mountain Pose)	1
3.b	Ardha Kati Chakrasana (Lateral Arc pose)	2
3.c	Padahastasana (Hand to foot pose)	1
3.d	Ardha Chakrasana (Standing Backward Bend or Half wheel pose)	1
3.e	Vrikshasana (Tree pose)	1
3.f	Utkatasana (with/without support) (Chair pose)	1
**4**	**Yogic Relaxation – Instant Relaxation Technique (3 minutes)**	
4.a	Bhujangasana Breathing (Cobra pose)	1
4.b	Shashankasana Breathing with Bhramari (Rabbit pose)	1
**5**	**Yogic Breathing (4 minutes)**	
**6**	**Kriya/Pranayama (4 minutes)**	
6.a	Slow phase Kapalabhati (Skull shinning Breath)	1
6.b	NadiShuddhi Pranayama (Alternate Nostril Breathing)	3
**7**	**Breath awareness (10 minutes)**	
**8**	**Yogic Relaxation – Quick Relaxation Technique (3 minutes)**	
	**Total**	**40 min**

Patients will undergo supervised yoga session for 18 weeks (5 days/week) at the oncology centre and home based. The intervention is tailored primarily to prevent chemobrain and cardiac dysfunction symptoms following chemotherapy.

The intervention comprises of asanas (postures), breathing exercises, pranayama (voluntarily regulated nostril breathing), mindfulness-based meditation, and imagery-based yogic relaxation techniques. We have primarily involved asanas that are balancing in nature as they are known to improve attention and memory with proven effects of stress reduction. Among pranayama (regulated nostril breathing), yogic breathing with breath-holding (Kumbhaka) after exhalation, Bramari (Humming bee sound) and Nadishuddi pranayama were involved. Kapalabhati is known to increase brain blood flow and improve cognition (44). These practices will be interspersed with Yogic relaxation techniques. All these practices were based on principles of enhancing attention, awareness, and relaxation to cope with stressful experiences faced by cancer patients during and after treatment.

Our yoga module encompasses balancing postures (Vrikshasana, Tadasana (centering awareness), padahastasana, ardha chakrasana, ardha kati chakrasana, bhujangasana and shashankasana with “m” kar chanting, followed by yogic breathing, kapalabhati, nadishuddhi and bramari pranayama. The literature review from yoga texts are appended as under.

The attentional cues facilitated by asanas would help in modulating attentional process involved in cognition and help modulate cognitive function and flexibility. Therefore asanas confer not only physical flexibility but also modulate cognitive flexibility. This is emphasised in the Patanjali yoga sutras in chapter 2, verse 48.

During pranayama, internalising the awareness of the breath, such as the motions of inhalation, exhalation, and the kumbhaka, will help calm down the mind and clear mind of all other distractions. This helps prepare the mind for concentration and attention, improving cognitive faculties.

Furthermore, these practices were shown to improve cognitive and cardiac functions in earlier studies conducted at the HCG cancer centre and various oncology centres across India (32, 45).

### Data collection

#### Assessment of cognitive functioning

Participants complete a neuropsychological test battery at four of the five study time points [baseline (pre-intervention), end of chemotherapy (post-intervention/primary endpoint), 16 weeks post-chemotherapy (first follow-up), and one-year post-baseline (second follow-up)]. The battery covered attention/working memory, verbal and visual memory, executive functioning, planning and processing speed. These cognitive domains were chosen because previous studies have shown that they are sensitive to the deleterious effects of chemotherapy (46). The tests are performed in the following order: Colour Trials Test conditions 1&2 (attention), Digit Span Test (Memory and Recall), Stroop Test (Executive functioning), Digit-Symbol Substitution Task (processing speed), Rey auditory Verbal Learning Test (Verbal Learning and memory), Tower of London (planning and executive functioning), and Visual Reproduction task (visual learning and memory). To minimize the effects of practice, different testing forms are used at different time points. The scheme of Neuropsychological test battery planned to use in the study is depicted in **Table 2**.

**Table 2:** Neuropsychological test battery.

Domain	Functions	Test	Source	Time Taken
Speed	Mental Speed	Digit Symbol Substitution	NIMHANS Neuropsychology Battery	5-7 minutes
Attention	Focussed Attention and Cognitive Flexibility	Colour Trails Test 1 & 2	NIMHANS Neuropsychology Battery	8-10 minutes
Executive Function/ Language	Phonemic Fluency	Controlled Oral Word Association Test	NIMHANS Neuropsychology Battery	5 minutes
	Semantic Fluency	Animals Names Test	NIMHANS Neuropsychology Battery	3 minutes
Executive Functions	Verbal Working Memory	Verbal N Back Test (1 back and 2 Back)	NIMHANS Neuropsychology Battery	5-7 minutes
	Response Inhibition	Stroop Test	NIMHANS Neuropsychology Battery	10-12 minutes
Visuo-Spatial	Visuo-Spatial Construction	Rey Complex Figure Test	NIMHANS Neuropsychology Battery	10-15 minutes
Learning and Memory	Visual Memory	Rey Complex Figure Test		
	Verbal Memory	Rey Auditory Verbal Learning Test	NIMHANS Neuropsychology Battery	15 minutes
Language	Speed Reading	Comprehension

Justification: In order to assess Cognitive functions, we will use both self-report instruments to assess cognitive impairment and also evaluate cognitive functions through a task performance-based tool. Earlier studies have shown problems with working memory, attention span, information processing and mental speed in breast cancer patients following six cycles of chemotherapy (47).

#### FACT Cog

Functional Assessment of Cancer Therapy Cognitive Function (FACT-Cog) is a self-report measure used to assess cognitive impairment and its effect on the patient’s quality of life. The FACT-Cog was the first patient-reported outcome measure evaluating cognitive impairment that was validated with cancer patients. It was derived from interviews with expert clinicians and oncology patient focus groups. This 37-item assessment tool allows patients to evaluate their memory, attention, concentration, language, and thinking skills. It consists of four subscales, assessing perceived Cognitive Impairments that occurred during the last seven days, on a 5- point Likert scale (from 0 “never” to 4 “several times a day”) (48) (49).

#### Heart rate variability (HRV)

The basal cardiac autonomic status will be evaluated using heart rate variability, which measures sympathetic or parasympathetic dominance. The Electrocardiogram (ECG) is recorded for 20 min, in which 5 min artefact free and resting segment will be used for analysis. Ag/AgCl solid adhesive pre-gelled electrodes (3M) will be used. The ECG will be acquired using an ambulatory ECG system (AD Instruments) at a sampling rate of 1024 Hz and will be stored for offline analysis. The data would be analysed with an advanced HRV analysis software program (LAB CHART PRO) that is inbuilt in the system.

#### Echocardiography

Echocardiography is a test that uses high-frequency sound waves (ultrasound) emitted by a transducer across the chest. The probe generates sound waves that “echo” back to the probe. These waves are transformed into images on a monitor. Echocardiography is widely available, relatively inexpensive and can assess diastolic function. Conventional echocardiography assesses structural and functional parameters, such as left ventricular (LV) ejection fraction (EF), fractional shortening (FS), as well as diameters and volumes (50, 51).

##### Serum brain-derived neurotrophic factor (BDNF)

Assessment takes place at four out of five-time points: baseline, end of chemotherapy, first follow-up, and second follow-up. 5 ml of whole blood will be collected in a heparinized tube using a vacutainer system at each time point. Then samples will be immediately centrifuged at 2500 rpm for 10-15 min. The serum will be stored at −80°C until analysis. The serum BDNF level will be quantified using an enzyme-linked immunosorbent assay (ab212166, Abcam, Cambridge, UK).

##### Serum cortisol

The serum cortisol level will be estimated using an enzyme-linked immunosorbent assay (ab108665, Abcam, Cambridge, UK). Assessment takes place at four of the five study time points: baseline, end of chemotherapy, first follow-up, and second follow-up. Changes in cortisol levels were observed in earlier studies with yoga intervention in breast cancer patients (52).

#### Functional MRI sequences

For f MRI: Each MRI scan session would consist of the image sequences listed below,

1. T2-weighted turbo spin echo sequence with TR/TE/number of excitations (NEX) =5600ms/100ms/1, field of view=220×220 mm2, slice thickness = 4mm with no interslice gap and number of slices=25.
2. High-resolution T1-weighted images will be acquired using a magnetization prepared rapid acquisition gradient-echo (MPRAGE) sequence (TR = 1900 ms; TE = 2.07 ms; inversion time = 900 ms; matrix size = 256×256; FOV = 256×256 mm; inversion time = 900 msec; flip angle (FA) = 9°; slice thickness = 1 mm; number of slices = 160).
3. Diffusion kurtosis imaging data will be acquired using an echo planar imaging with twice-refocused spin echo pulse sequence in the axial plane (TR = 7300 msec; TE = 138 msec; FA = 90°; matrix-size = 128 × 128; FOV = 230 × 230 mm; slice thickness = 4.5 mm; 30 slices; no interslice-gap; spatial resolution = 1.797×1.797×4.5mm; diffusion directions = 30; b values = 0, 1000, 1500 and 2000 s/mm2).
4. Functional brain volumes will be acquired using an echo-planar T2* -weighted imaging sequence. Each volume consisted of 30 interleaved 5-mm thick slices without inter-slice gap (TE = 30 ms, TR = 2000 ms, FOV = 240 mm, flip angle = 90°, voxel size = 3.75× 3.75 ×5 mm3). Total scanning time was 410 seconds (205 brain volumes).
5. MRS will be obtained using Point Resolved Spectroscopy sequence (PRESS) with acquisition parameters: TR/TE = 2000ms/35ms; 2048 spectral points; 1200 Hz spectral Bandwidth and 256 averages. Unsuppressed water (with 16 averages) spectra will also be acquired immediately before the water-suppressed metabolic acquisition and was used for spectral quantifications. For frontal region, a voxel of 12×12×12mm3 will be placed in the frontal region of the brain. For anterior cingulate (ACC), a voxel of 10×10×5 will be positioned in the left ACC. The resting scan will allow us to image the intrinsic connectivity pattern.

#### Quality of life as measured by self-report EORTC QLQ C30

European Organization for the Research and Treatment of Cancer-Quality of Life (EORTCQoL C30 questionnaire version 3) will be used to assess health-related quality of life (53). This 30-item questionnaire examines global health status, physical, role, emotional, cognitive, and social functioning (with high scores indicating a high quality of life) and cancer-related symptomatology. Its validity, reliability, and sensitivity in cancer patients are well established (31, 53). Assessments will be carried out at five-time points: Baseline, pre-chemotherapy, during chemotherapy, post-chemotherapy, 16-weeks post-chemotherapy and 1-year post baseline.

#### Stress as measured by self-report perceived stress scale (PSS)

The PSS is a ten item-question scale typically used to estimate perceived stress or how individuals view their current situations as stressful. The reliability (internal reliability) of the PSS instrument is robust, ranging from 0.84 to 0.86, and its validity ranges from 0.31 to 0.76 when correlated with measures of physical and mental symptoms (54). In the proposed study assessment will be carried out at all five-time points

##### Psychological wellbeing

Questionnaire Ryff developed by Carol Ryff (55) will be used to assess psychological wellbeing at all the five time points of the study (Baseline, pre-chemotherapy, during chemotherapy, post-chemotherapy, first follow-up and second follow-up). This scale measures the elements of autonomy, environmental mastery, personal growth, positive interpersonal relationships, life purpose, and self-acceptance. Furthermore, this scale has been used widely to assess psychologic wellbeing in cancer patients (56).

##### Statistical analyses

Data will be analyzed using IBM SPSS (Version 22.0 for Windows). Sociodemographic and medical characteristics will be characterized using descriptive statistics. To evaluate attrition bias, sociodemographic and medical factors at baseline will be compared between participants who completed all assessments and those who dropped out. Under the “missing at random” assumption, characteristics usually linked with attrition (e.g., age, chemotherapy duration) will be included as covariates in all analyses.

The data will be analysed for intention to treat analysis accounting for dropouts in the groups. Data will be analysed at baseline (before chemotherapy starts), after three and six cycles of chemotherapy. We will; also try to analyse six months and one-year follow-ups. A repeated measures ANOVA will be done using *post hoc* Bonferroni correction. A p-value ≤ 0.008 will be considered significant (95% confidence interval) for a 2 group assignment over three-time points (interaction effects). Secondary analyses will be carried out to determine the bivariate relationships between predictor and outcome variables. Further regression analyses will be done to understand the mediation and moderation effects if any.

#### Monitoring

The progress of the study will be monitored by an internal project evaluation committee (IPEC) that meets periodically. IPEC will be responsible for trial data monitoring and quality assurance. The committee holds biannual meetings to ensure the trial’s proper conduct and safety and to offer/propose trial-related suggestions/modifications. In addition, the committee evaluates descriptive/interim reports that detail recruiting and enrolment numbers, sample characteristics, primary and secondary outcomes, and adverse events.

## Discussion

This study has planned to address the issues, extremely relevant in the area of breast cancer research and clinical management. There has been enhanced recognition of the potential of yoga as add on intervention for treatment induced adverse effects in breast cancer patients. This study will be the first of its kind to examine the efficacy of yoga on CRCI and CRCD in a controlled randomized control trial design.

Drugs that are a standard part of the chemotherapy regimen for breast cancer, such as Fluorouracil, Methotrexate, Paclitaxel and Taxanes, are recognized as neurotoxic and have a direct dose-response effect on cognition (57).

The yoga intervention module intended to use in this study has been shown to improve various aspects of cognitive and cardiac function with improved clinical outcomes in the non-cancer and cancer population (32, 39). Yoga has been shown to improve cognition in a variety of ways. First, yoga helps reduce stress and improve mood that affects cognitive functioning (36, 58). Secondly, yoga programs have been shown to improve attention span and the ability to shift attention and working memory on various neuropsychology tests (59). The attentional and alertness systems are critical components necessary for all aspects of cognition, including memory and language. Attention may be more amenable to change than other aspects of cognition (60, 61). Internalizing one’s awareness (attention) or mindfulness is an essential component of yoga that is known to help practitioners recognize stressful stimuli and modulate or slow down reactions to stressful stimuli, thereby helping them cope with stress. Studies have shown that yoga components such as asanas, pranayama, meditation, and relaxation improve attentional abilities in cognitive function (62). Third, both slow and fast breathing affect vagal afferents through their action on stretch receptors in the lung tissue and diaphragm and increase parasympathetic tone akin to relaxation response that calms down the mind (59). An earlier study on healthy volunteers saw a significant reduction in perceived stress and improvement in the following cognitive domains: attention, visuomotor speed and memory retention capacity in fast and slow pranayama groups (36). Fourth, asanas or postures facilitate physical training, strength, cardiorespiratory effort, aerobic capacity and balance, attentional faculties, and improved cognitive performance (63, 64). Studies have shown that cognitive spatial processing may rely on neural mechanisms that are also required to regulate posture; therefore, having balancing postures may help facilitate attention and spatial memory (62). Preliminary studies show the effects of a similar yoga program on cognitive functions in cancer patients (65, 66). A recent meta-analysis of yoga and cognition has shown benefits in improving cognitive function in adults and elderly populations showing substantial improvement in attention processing, memory, and executive functions as acute effects following yoga (67).

In current oncology practice, the ‘cardiovascular’ impact of cytotoxic therapies is estimated solely by changes in resting left ventricular ejection fraction (LVEF) which in turn is load-, rate-, and contractility-dependent and acute declines in myocardial function can be initially compensated to maintain cardiac output (68). Consequently, left ventricular dysfunction is a late marker, becoming evident after significant myocardial damage has already occurred resulted in need of alternative tools to identify patients in early stages who are at high risk for adverse cardiovascular impacts. Hence the current study used HRV along with echocardiography to detect CAD at a sub clinical stage. The study is the first to examine this neurocognitive changes correlating with CAD and serum cortisol. Another considerable strength of the trial is its multi-faceted assessment in understanding the impact of yoga intervention on brain structure (MRI) and functioning (BDNF) (fMRI), with neurocognitive functioning measures, cardiac vagal tone, and quality of life. Furthermore, these extensive outcome assessments on several levels involving various tools will allow researchers to investigate the relationship between subjective scales and objective indicators.

Hence, owing to the acute need for developing disease-modifying therapies that may improve and/or relieve symptoms associated with chemotherapy. The results from this study could have an immediate effect (yoga as an adjuvant treatment for breast cancer) as well as long term clinical implications in modulating cognitive function (development of novel yoga techniques based on understanding the mechanism of yoga). Moreover, the study also has important theoretical implications by shedding light on the role of yoga in modifying the behaviour of patients with Breast cancer receiving chemotherapy. In conclusion, this study will be the first to answer the leading edge questions in yoga and breast cancer research addressing CRCI and CRCD collectively.

## Strength of the study

The trial (1) recruits an adequate sample size to detect clinically meaningful changes in cognitive and cardiac functioning, (2) includes both self-report and objective measures of cognitive and cardiac functioning, and (3) assesses outcomes at five timepoints across a 1-year period. Finally, attendance and adherence data to the yoga intervention will provide helpful information regarding women’s ability and willingness to participate in the intervention that lasts five days per week throughout chemotherapy.

## Conclusion

The study outcome believes that yoga could be a feasible and effective approach for preventing and/or reducing CRCI and CRCD, improving quality of life, and enhancing supportive care for breast cancer patients undergoing chemotherapy. There is currently no pharmacological interventions available to prevent or alleviate these cognitive difficulties arise due to chemotherapy in cancer patients. Yoga, a promising non-pharmacological intervention, could be beneficial in addressing this chemotherapy induced cognitive decline and cardiac dysfunction and improving overall QOL. If the current study shows yoga to be beneficial, patients with breast cancer could be offered an evidence-based intervention to help them cope with the side effects of chemotherapy (cognitive and cardiac issues) while also improving their quality of life. In addition, we anticipate that the findings of this study will be useful for chemotherapy-exposed individuals with other solid cancer types who have cognitive and cardiac issues. Furthermore, this study might also positively influence the quality of life of the growing community of (breast) cancer survivors. Hence, the discoveries that are intended to achieve through this trial could substantially contribute to the current state of knowledge and help develop future yoga interventions to prevent and/or mitigate CRCI and CRCD with added health benefits.

## Limitations

A potential limitation of this protocol is that participants and the yoga therapist cannot be blinded because it is challenging to conduct blinding in non-pharmacological studies. Therefore, performance bias may be unavoidable. Furthermore, the study lacks an active control group (an exercise group). Also, the present study might hamper generalisability to a broader patient population who experience cognitive problems during cancer chemotherapy.

## Risk of yoga practice

We anticipate only negligible physical risks associated with yoga, as it involves low-impact, highly controlled movements confined to an individual’s range of motion and will be taught and supervised by a skilled yoga therapist. However, the relaxation techniques in the same session could be adequate to overcome these issues. Furthermore, even if there were significant physical limitations during the yoga session, appropriate modifications would be made by the yoga therapist to reduce the participant’s physical discomfort, and further medical assistance will be provided.

## Ethics statement

The studies involving human participants were reviewed and approved by HCG-Central Ethics Committee (EC/434/19/01) National Institute of Mental Health and Neurosciences (No.NIMH/DO/ETHICS SUB-COMMITTEE BS&NS 9th Meeting/2018). The patients/participants provided their written informed consent to participate in this study.

## Authors contributions

This work is a collaborative effort in which all authors have an equal contribution. RR, AR, TS, KU, and GI contributed to the conception and design of the study as well as writing and editing the manuscript. KN, SB, and SKB contributed to data acquisition and teaching yoga to the participants. Oncologist SP helped with clinical evaluation and referring patients to the study. JR contributed to designing the neuropsychological test battery and evaluation. Overall, all authors read, revised, and approved the final manuscript.

## Funding

The trail is funded by the Extramural Research Scheme of Ministry of AYUSH (Z.28015/62/2016-HPC(EMR)-AYUSH-B) and Department of Science & Technology under the scheme: Science and Technology of Yoga and Meditation (SATYAM) (SR/SATHYAM/389/2015).

## Conflict of interest

Authors AR, KN, SB, SKB, SP and RR were employed by HealthCare Global.

The remaining authors declare that the research was conducted in the absence of any commercial or financial relationships that could be construed as a potential conflict of interest.

## Publisher’s note

All claims expressed in this article are solely those of the authors and do not necessarily represent those of their affiliated organizations, or those of the publisher, the editors and the reviewers. Any product that may be evaluated in this article, or claim that may be made by its manufacturer, is not guaranteed or endorsed by the publisher.
